# Protocol for a systematic review and meta-analysis of research on the associations between workplace bullying and sleep

**DOI:** 10.1186/s13643-018-0898-z

**Published:** 2018-12-13

**Authors:** Morten Birkeland Nielsen, Ståle Pallesen, Anette Harris, Ståle Valvatne Einarsen

**Affiliations:** 1 National Institute of Occupational Health, Pb 5330 Majorstuen, N- 0304 Oslo, Norway; 2 Department of Psychosocial Science, University of Bergen, Bergen, Norway

**Keywords:** Harassment, Aggression, Work, Health, Distress, Synthesis

## Abstract

**Background:**

Existing evidence on the association between exposure to bullying and sleep is limited and inconclusive. The aims of this planned systematic review and meta-analysis are therefore (1) to determine whether exposure to workplace bullying is related to changes in sleep function and (2) to establish mediating and moderating factors that govern the relationship between bullying and sleep.

**Methods:**

A systematic review and meta-analysis will be conducted. Electronic databases will be searched using predefined search terms to identify relevant studies. Eligible studies should report empirical findings on the association between exposure to workplace bullying and at least one indicator of sleep. Primary observational studies with cross-sectional or prospective research design, case-control studies, and studies with experimental designs will be included. Qualitative interviews and case studies will be excluded. The methodological quality of the included studies will be assessed with a previously established checklist for studies on workplace bullying. The quality of evidence for an association between bullying and sleep problems will evaluated in accordance with the GRADE system. A random effects meta-analysis will be conducted with the Comprehensive Meta-Analysis software, version 3.

**Discussion:**

This review and meta-analysis will be among the first to systematically explore and integrate the evidence available on the association between exposure to bullying and sleep, as well as on the mediating and moderating factors that can govern this associations. By gathering and summarizing information about potential factors that can explain when and how bullying is related to sleep, the findings from this study will provide directions for future research and provide practitioners and clinicians with an understanding about the nature and consequences of workplace bullying and point to directions for relevant interventions.

**Systematic review registration:**

The protocol has been registered at the International Prospective Register of Systematic Reviews (PROSPERO; registration number: CRD42018082192).

**Electronic supplementary material:**

The online version of this article (10.1186/s13643-018-0898-z) contains supplementary material, which is available to authorized users.

Quality of sleep is highly important with regard to everyday functioning, mental and physical health, and for job performance [1]. A 2011 US study estimated the socioeconomic costs of troubled sleep to be in the area of 63 and 91 billion dollars per year [2]. To reduce these costs, knowledge about the antecedents and risk factors for sleep problems is therefore of major significance. To this date, we know that physical and psychosocial working conditions are associated with a range of negative outcomes, including sleep problems [3]. Although negative social interactions at the workplace, such as bullying, may be especially distressing for those exposed [4, 5], previous research on psychosocial work environment factors and sleep has mainly been limited to examining the impact of job demands and control [6–8]. Consequently, there is a shortage of knowledge about how and when social problems, such as exposure to workplace bullying, influence sleep. Workplace bullying, defined as a situation wherein an employee persistently and systematically is exposed to harassment and mistreatment at work and wherein this employee finds it difficult to defend him- or herself against this prolonged and unwanted treatment [9], has been established as a precursor to a range of health complaints including depression and anxiety [10–12], somatic problems [11], and even symptoms of posttraumatic stress [13]. Bullying has also been highlighted as a potential cause of sleep problems [5, 14, 15]. For instance, in a mixed method study on workplace bullying among university employees, insomnia was reported by practically all cases interviewed [16]. In a study among victims of bullying, findings revealed a high prevalence of sleep problems, specifically difficulties falling asleep, interrupted sleep, fatigue during the day, and early morning awakening [17]. Still, existing evidence is limited, and in a meta-analysis published in 2012, only four studies, encompassing 14,584 respondents, were included [10]. Going against expectations about an association between bullying and sleep problems, a non-significant Pearson correlation of .10 (95% CI = − .29–.45) was reported in the meta-analysis. It should be noted that this latter meta-analysis focused on outcomes of bullying in general and did therefore not include a systematic review of the literature on bullying and sleep.

The Cognitive Activation Theory of Stress (CATS) [18] has been suggested as a theoretical framework for how exposure to bullying could influence sleep [4]. According to CATS, cognitive activation is a key factor in the cycle of emotional and physiologic arousal on sleep, and an extensive body of research has shown that cognitive activation is associated with persistent high stress levels and pathology, such as decreased sleep quality, increased cortisol levels, elevated heart rate, and increased mortality [18, 19]. In short, persistent exposure to stressors leads to increased arousal. This increase in arousal as a response to stressful situations may prolong physiological activation which subsequently can be manifested through difficulties in sleep initiation or returning to sleep after awakenings during the night. However, there may be individual differences in responses to bullying, and some workers may be more able to cope with the exposure compared to others [20]. It is also possible that different workers respond with different profiles to stressors [21]. Overall, this suggests that the impact of workplace bullying on sleep should be both mediated (activation) and moderated (individual differences) by other variables.

To add to our knowledge about the potential impact of bullying on sleep, this planned meta-analytic study will provide a systematic review of all available research literature on the associations between the variables. The first aim is to determine whether exposure to workplace bullying is related to levels of sleep among employees. The overall magnitude of this association will be established by means of a meta-analytic synthesis. Theoretically, bullying may have indirect (mediated), conditional (moderated), and reverse associations with sleep parameters. In addition, sleep may function as a mediator between bullying and other indicators of health and well-being. A secondary aim of the study is therefore to review and summarize research on such mediating and moderating factors affecting the relationship between workplace bullying and sleep. Hence, this study will extend existing reviews on bullying and sleep by including a larger number of studies, thus increasing the statistical power in the meta-analysis, and by examining moderating and mediating factors that determines when and how exposure to bullying relates to sleep.

## Methods

The following protocol has been written according to the MOOSE Guidelines for Meta-Analyses and Systematic Reviews of Observational Studies and the PRISMA-P (Preferred Reporting Items for Systematic Reviews and Meta-Analyses) guidelines [22, 23]. The PRISMA-P checklist is given in the Additional file 1. The protocol has been registered at the International Prospective Register of Systematic Reviews (PROSPERO; registration number: CRD42018082192).

The proposed review and meta-analysis is part of a larger project entitled “Bullying in the workplace: from mechanisms and moderators to problem treatment.” The aims of that project are (1) to improve our understanding of the workplace bullying phenomenon through determining mechanisms (mediating and moderating factors) that influence and explain how and when workplace bullying occurs, develops, and impacts those targeted and (2) to provide information that can be used to develop sound and effective interventions and rehabilitation approaches for targeted individuals and organizations.

## Data sources search terms and search strategy

This literature review and meta-analysis will be based on systematic searches in multiple literature databases, including Medline/Pubmed, Proquest, Web of Science, Taylor & Francis Online Journals, PsychInfo, and Wiley Online Library. Additional searches will be performed in Scopus and Google Scholar. All search terms are included in Table 1. Systematic searches will be conducted by combining every possible combination of three categories of keywords. Reference lists of key full text articles included in the review will be checked to identify any potentially eligible studies. The searches will not be limited by historical time-constraints. The systematic procedure substantiates that the literature search comprises all published studies on the relationship between workplace bullying and different sleep parameters. The search strategy is considered as adequate to reduce the risk of selection and detection bias. The search results will be exported to Endnote where duplicates are excluded. Included studies will be manually screened in order to select other relevant studies.

**Table 1:** Search terms used in literature search

Category 1	Category 2	Category 3
Work*	Bullying	Sleep*
Job	Mobbing	Insomnia
Occupational	Victimization	Awakening
Employee	Emotional abuse	Asleep
Worker	Incivility	Circadian
	Psychological aggression	Hypersomnia
	Mistreatment	Parasomnia
	Ostracism	Somnambulism
	Exclusion	Nightmare
	Undermining	Napping
	Harassment	Fatigue
		Dreams
		Apnea
		Polysomnography
		Actigraphy
		Somniphobia

Work* captures “workplace” “working,” etc. Sleep* captures “sleep problems,” “sleep disorders,” “sleep complaints,” “sleepiness” etc.

## Inclusion and exclusion criteria

Eligible studies should report empirical findings on the association between exposure to workplace bullying (or any overlapping concept) and an indicator of sleep (e.g., disturbed sleep, early awakening, etc.). Primary observational studies with cross-sectional or prospective research design, case-control studies, and studies with experimental designs will be included. Cross-sectional data will be used to determine the magnitude of the association between bullying and sleep, whereas prospective data will be used to determine directions of associations. As associations based on prospective data are dependent upon the utilized time-lag between measurement points [24], it is important to also include cross-sectional data. Qualitative interview studies, single-case studies, and series of single-case studies will not be included in the meta-analysis. To be included in the meta-analytic part of the study, studies should provide the zero-order associations between bullying and sleep or provide sufficient information for these associations (effect sizes) to be calculated. Studies lacking this information or reported effect sizes that could not be transformed into odds ratios will be excluded from the meta-analyses. To avoid double-counting data, the sample in a given study should not have been used in a previous study of those included in the review. In cases with overlap, we will use data from the largest sample. The review will be limited to articles published in peer-reviewed journals in English, German, French, or the Scandinavian languages (Danish, Norwegian, and Swedish). Hence, this will be a review of published peer review studies only. Accordingly, data based on conference abstracts, dissertations, and gray literature (e.g., reports, etc.) will not be included. As a first step, relevant articles will be considered on the basis of their title and abstract. At the second step, full-text versions of selected papers will be examined and assessed with regard to effect sizes and methodological quality.

A professional librarian will conduct the search. The primary investigator will oversee the search strategy and remove duplicates using Endnote X7. Following the above inclusion and exclusion criteria, two reviewers without consideration for the results will perform assessment of studies for potential inclusion independently. Any differences in opinions will be resolved through discussion until a consensus is reached. A third reviewer may be consulted if necessary. This process ensures that bias is minimized when deciding whether or not to include or exclude certain studies. The two reviewers will independently conduct the data extraction from each study using a pre-defined data extraction sheet. Following the description by Lipsey and Wilson [25], the coding form will assess information about bullying and sleep, demographic characteristics of participants (age, gender, job type, employment status, educational level, etc.), study characteristics (country of origin, sample size, effect sizes, response rate, year study published, sampling method, measurement inventories, etc.), and other relevant variables (health indicators, other exposures).

## Participants

The study population will be adults (18 years or older) with a current or previous employment in a full or part-time position. No restrictions will be placed on participants’ gender, ethnicity, or other demographic characteristics. Since the aim of the study is to determine associations between bullying and sleep, indicators of mental and somatic health complaints will be recorded and used as correlates and/or moderators in meta-analyses (conditioned by enough relevant studies). A minimum of two studies is considered sufficient to perform a meta-analysis [26].

## Assessment of methodological quality (risk of bias)

As displayed in Table 2, the methodological quality of the included studies will be assessed with an adapted version previously established checklist for research on workplace bullying comprising 14 items related to sampling, representativeness, measurement issues, and confounders [27]. This checklist comprises selected and adapted items from the Risk of Bias Assessment Tool for Nonrandomized Studies [28] and the Quality Assessment Tool [29]. The quality of the reviewed studies will be scored on a scale from 0 (lowest possible quality) to 13 (highest possible quality). Kappa will be calculated to quantify the level of inter-rater agreement.

**Table 2:** Checklist for the assessment of the methodological quality of the reviewed studies

		Points
Part 1. Sampling and representativeness		
1. Sampling method		
A	Non-probability sampling (including: purposive, quota, convenience and snowball sampling)	0
B	Probability sampling (including: simple random, systematic, stratified g, cluster, two-stage and multi-stage sampling)	1
2. Was the response rate reported?		
A	Not reported	0
B	Response rate below 50%	0
C	Response rate at 50% or above	1
3. Are the individuals selected to participate in the study likely to be representative of the target population?		
A	No	0
B	Yes	1
4. Selection bias: Is there a risk of selection bias caused by the inadequate selection of participants		
A	High risk	0
B	Low risk	1
5. Is the sample size adequate for establishing relationships (assumption of statistical power)		
A	No	0
B	Yes	1
Part 2. Measurement and confounders		
6. How was workplace bullying measured?		
A	Self-labeling without definition of the bullying concept	0
B	Self-labeling with a definition of the bullying concept	1
C	Behavioral checklist (e.g., NAQ, LIPT)	1
7. How was sleep (complaints) assessed?		
A	Self-report	0
B	Objective measurement	1
8. Performance bias: Is there a risk of performance bias caused by the inadequate measurement of exposure		
A	High risk	0
B	Low risk	1
9. Are the statistical methods appropriate for the study design?		
A	No/Cannot tell	0
B	Yes	1
10. Were meaningful demographic covariates included?		
A	No	0
B	Yes	1
11. Were other work factors adjusted for?		
A	No	0
B	Yes	1
12. Is the study design cross-sectional or prospective (with time-lag)?		
A	Cross-sectional	0
B	Prospective	1
13. Was previous sleep (complaints) adjusted for in prospective analyses?		
A	No	0
B	Yes	1
14. Confounder bias: Is there a risk of bias caused by the inadequate confirmation and consideration of confounding variable		
A	High risk	0
B	Low risk	1

The quality of evidence for an association between bullying and sleep problems will be evaluated in accordance with the GRADE system [30]. This system grades quality of evidence at four levels: high (4), moderate (3), low (2), and very low (1). For high evidence, the requirements are a randomized, double-blinded study design with no selection biases. For observational studies, moderate evidence, i.e., exceptionally strong evidence from unbiased studies, is considered the strongest possible level of proof for an association.

## Meta-analytic approach

The meta-analysis will be conducted with the Comprehensive Meta-Analysis (version 3) software developed by Biostat [31]. Odds ratio (OR) with 95% confidence intervals (95% CI) will be reported as an overall synthesized measure of effect size. The mean of the combined effect sizes will be calculated in studies where several effect sizes were reported from the same sample (e.g., models with different control variables). An overall estimate will be calculated for studies with overlapping samples. In studies reporting effect sizes from independent subgroups (e.g., moderators), each subgroup will be included as a unique sample in the meta-analysis. Moderation analyses will also be used to compare associations from cross-sectional and prospective data. In contrast to some other meta-analytic methods, such as the Hunter and Schmidt approach [32], which weights studies by sample size, the Comprehensive Meta-analysis program weights studies by inverse variance. Inverse-variance weighting is a method of aggregating two or more random variables where each random variable is weighted in inverse proportion to its variance in order to minimize the variance of the weighted average. The inverse variance is roughly proportional to sample size, but is a more nuanced measure, and serves to minimize the variance of the combined effect [33].

As the individual studies included cannot be expected to come from the same population of studies, pooled mean effect size will be calculated using the random effects model. Such effects models are thus recommended when accumulating data from a series of studies where the effect size is assumed to vary from one study to the next and where it is unlikely that studies are functionally equivalent [33]. Random effects models allow statistical inferences to be made to a population of studies beyond those included in the meta-analysis [34]. The *Q*_within_-statistic will be used to assess the heterogeneity of studies. A significant *Q*_within_-value rejects the null hypothesis of homogeneity. An *I*^2^ statistic will be computed as an indicator of heterogeneity in terms of percentages. Increasing values show increasing heterogeneity, with values of 0% indicating no heterogeneity, 50% indicating moderate heterogeneity, and 75% indicating high heterogeneity [35]. The “one-study-removed” procedure will be used as a sensitivity analysis to determine whether the overall estimates between bullying and sleep are influenced by outlier studies. Using this approach, effect sizes that fell outside the 95th confidence interval of the average effect size will be considered as outliers. Four indicators of publication bias are to be examined: funnel plot, Rosenthal’s Fail-Safe N, Duval and Tweedie’s trim and fill procedure, and Egger’s regression intercept [36].

## Discussion

This planned review and meta-analysis will systematically explore the evidence available on the association between exposure to bullying and sleep. By gathering and summarizing information about potential mediating and moderating factors that can explain when and how bullying is related to sleep, the findings from this study will provide directions for future research and provide practitioners with an understanding about the nature and consequences of workplace bullying. This knowledge can be used to develop stronger countermeasures and interventions. Both bullying and sleep can be considered as modifiable factors that, if assessed and promptly recognized, can be addressed, potentially preventing the development of further health problems.

## Limitations

As data will be extracted using full-text articles only, and excluding data from gray literature, this review will build on published studies and doctoral dissertations exclusively, whereas unpublished studies and non-peer reviewed literature (e.g., reports) are to be excluded. Although it has been suggested that researchers should aim at including unpublished literature in meta-analyses and systematic reviews, the inclusion of data from unpublished studies can itself introduce bias [37]. First of all, the unpublished studies that can be located are likely to be an unrepresentative sample of all unpublished studies. For instance, the identification of unpublished studies may depend on the willingness of investigators of unpublished studies to provide data. This may again depend upon the findings of the study, with more favorable results being provided more readily [37]. Secondly, unpublished studies may be of lower methodological quality than published studies. In a study of 60 meta-analyses that included published and unpublished studies it was found that unpublished studies were less likely to conceal intervention allocation adequately and to blind outcome assessments [38]. As the planned review will be based on a comprehensive literature search of studies published in peer reviewed journals, the scientific quality of the included studies should be ensured while they at the same time also should be representative for the published literature on workplace bullying and sleep. Furthermore, the robustness of the findings will also be indicated by publication bias analyses.

It is likely that most associations reported in primary studies will be based on self-report data based on the self-administered questionnaires. This kind of data is prone to be influenced by common method bias as well as response set bias such as expectations, previous experiences, or health status. This may cause both non-differential and differential misclassification, resulting in under- and overestimations of effects [39]. However, sleep data based on actigraphy and polysomnography can be regarded as unbiased.

The meta-analysis will include studies with cross-sectional designs, and the aggregated effect sizes will therefore not account for the cause and effect relationship between the included variables. However, separate analyses will be conducted for studies based on time-lagged data in order to determine direction of associations over time.

## Ethics and dissemination

Ethical approval is not required for this systematic review and meta-analysis as only a secondary analysis of data already available in scientific databases will be conducted. The results of this review will be submitted for peer-reviewed publication and will be presented at relevant conferences.

## Review status

The project team has commenced searching relevant studies in the relevant databases. This review is expected to be complete by April 2019.

## Additional file

Additional file 1: PRISMA-P 2015 checklist. (DOCX 30 kb)
